# Therapeutic Attributes of Endocannabinoid System against Neuro-Inflammatory Autoimmune Disorders

**DOI:** 10.3390/molecules26113389

**Published:** 2021-06-03

**Authors:** Ishtiaq Ahmed, Saif Ur Rehman, Shiva Shahmohamadnejad, Muhammad Anjum Zia, Muhammad Ahmad, Muhammad Muzammal Saeed, Zain Akram, Hafiz M. N. Iqbal, Qingyou Liu

**Affiliations:** 1 State Key Laboratory for Conservation and Utilization of Subtropical Agro-Bioresources, Guangxi University, Nanning 530005, China; saif_ali28@yahoo.com; 2 School of Medical Science, Gold Coast Campus, Griffith University, Southport, QLD 4222, Australia; zain.akram@griffithuni.edu.au; 3 Department of Clinical Biochemistry, School of medicine, Tehran University of Medical Sciences, Tehran 14176-13151, Iran; shiva_shahmohamadnejad@yahoo.com; 4 Enzyme Biotechnology Laboratory, Department of Biochemistry, University of Agriculture, Faisalabad 38040, Pakistan; rmazia@hotmail.com (M.A.Z.); muzammalsaeed143@gmail.com (M.M.S.); 5 Faculty of Veterinary Sciences, Shaheed Benazir Bhutto University of Veterinary and Animal Sciences (SBBUVAS), Sakrand 67210, Pakistan; mahmad118@yahoo.com; 6 Tecnologico de Monterrey, School of Engineering and Sciences, 64849 Monterrey, Mexico; hafiz.iqbal@tec.mx

**Keywords:** endocannabinoid system, CB_1_ and CB_2_ receptors, cannabis, cancer, immunosuppressive

## Abstract

In humans, various sites like cannabinoid receptors (CBR) having a binding affinity with cannabinoids are distributed on the surface of different cell types, where endocannabinoids (ECs) and derivatives of fatty acid can bind. The binding of these substance(s) triggers the activation of specific receptors required for various physiological functions, including pain sensation, memory, and appetite. The ECs and CBR perform multiple functions via the cannabinoid receptor 1 (CB_1_); cannabinoid receptor 2 (CB_2_), having a key effect in restraining neurotransmitters and the arrangement of cytokines. The role of cannabinoids in the immune system is illustrated because of their immunosuppressive characteristics. These characteristics include inhibition of leucocyte proliferation, T cells apoptosis, and induction of macrophages along with reduced pro-inflammatory cytokines secretion. The review seeks to discuss the functional relationship between the endocannabinoid system (ECS) and anti-tumor characteristics of cannabinoids in various cancers. The therapeutic potential of cannabinoids for cancer—both in vivo and in vitro clinical trials—has also been highlighted and reported to be effective in mice models in arthritis for the inflammation reduction, neuropathic pain, positive effect in multiple sclerosis and type-1 diabetes mellitus, and found beneficial for treating in various cancers. In human models, such studies are limited; thereby, further research is indispensable in this field to get a conclusive outcome. Therefore, in autoimmune disorders, therapeutic cannabinoids can serve as promising immunosuppressive and anti-fibrotic agents.

## 1. Introduction—Problem and Opportunities

The endocannabinoid system (ECS) is composed of endocannabinoids (ECs), associated receptors of cannabinoid, and metabolizing enzymes. ECs are endogenous lipid-based retrograde neurotransmitters in a biological system. They are bound to cannabinoid receptors (CBR), and cannabinoid receptor proteins are expressed via the vertebrate central nervous system (CNS) and peripheral nervous system. Cannabinoids are known as a group of terpene phenolic compounds and found in the *Cannabis sativa* (marijuana plant). Commonly, three types of cannabinoids are identified: (i) phytocannabinoids observed distinctively in the *Cannabis sativa* plant, (ii) endogenous cannabinoids found in mammals (i.e., humans and animals), and (iii) laboratory-based cannabinoids (i.e., synthetic) [1,2,3,4]. Living organisms respond to complex stimuli, and an evolutionarily conserved form of ECS exists from plants to mammals. The cannabinoids (over 80) are produced from *Cannabis sativa.* Their broad-spectrum characterization classifies them as an assembly of substances with a substantial structural correlation with Delta-9-tetrahydrocannabinol (Δ^9^-THC) and binds to the CBR. Marijuana is a primary active component of *Cannabis sativa*, which has been found highly effective to treat wide-ranging syndrome in patients with cancers, AIDS, CNS disorders (i.e., multiple sclerosis). Moreover, glaucoma is also included in the list of treatments by those who believed in the medicinal aspects of marijuana [5,6,7,8,9]. The chemistry of these substances shows various classes of particular chemicals, such as the close structural similarity of classical cannabinoids to the Δ^9^-THC, non-classical categorized cannabinoids, the aryl sulphonamides, the ECs related eicosanoids, the aminoalkylindoles, and the quinoles [10,11]. There are additional compounds not categorized into these standard classes due to specific physicochemical characteristics, even though those exhibit the binding affinity to CBR (CB_1_ and CB_2_). Multi-dimensional characterization of marijuana on their potential medical effects can be selected during the evaluation parameters of marijuana and cannabinoids concurrence of specific human diseases, with fewer side effects. In the previous decades, the endocannabinoid pathway and the physiological impacts of cannabinoids have been studied extensively. Cannabinoids exhibit immunomodulatory effects, and their application, along with prospective roles as an autoimmune or inflammatory therapy, has widely been explored [12].

This review comprises the immunomodulatory and therapeutic potential of the ECS against different autoimmune disorders and other disease conditions, emphasizing cancer treatments and their biomedical perspectives in the 21st century. Particularly, we focused on organizing the research studies of ECs as promising treatment options for multiple sclerosis, type-1 diabetes mellitus, anti-inflammatory, and anti-fibrotic agents, rheumatoid arthritis (RA), and ameliorating neuropathic pains. The effective utility of practical therapeutics against cancer and tumor conditions has also been explained in later sections. The compilation of this updated information will be useful for medical researchers and professionals, pharmacists, and research scholars for designing and developing effective drug(s) strategies and biomedicines to counter various present-day human diseases and disorders.

## 2. The Endocannabinoid System

An ECS comprises endogenous ligands, associated CBR (particularly CB_1_ and CB_2_), and metabolic enzymes. Endocannabinoid receptors were named CBR after the recognition of endogenous ligands. The ECs are obtained from the membrane that is composed of phospholipids. Therefore, they are known as bioactive lipid mediators. After the identification of the first lipid mediator, arachidonoyl-ethanolamide (AEA) of the ECS, (also known as anandamide) [13,14,15], different biomolecules associated with this family were discovered. The most vital molecules are 2-arachidonoyl-glycerol (2AG) and its isomer 1AG among monoacyl-glycerols; palmitoyl-ethanolamide (PEA); oleyl-ethanolamide (OEA), and the N-acyl-ethanolamides [16,17,18]. The cannabinoid receptor type-1 and type-2, both 2AG and AEA are engaged in different biological functions; however, the AEA metabolism and attachment of the peroxisome proliferator-activated receptor-α is influenced by PEA and OEA [8,19,20,21,22,23]. These all biomolecules are explained in detail in the endocannabinoid related compounds. Partial or full agonists of CB_1_ receptors in terms of anandamide depend on tissue and biological response type. However, CB_2_ receptors can attach but have an intermittent effect and can perform like an antagonist [19,20,24].

### 2.1. Cannabinoids Receptor Agonists

**Figure 1:**
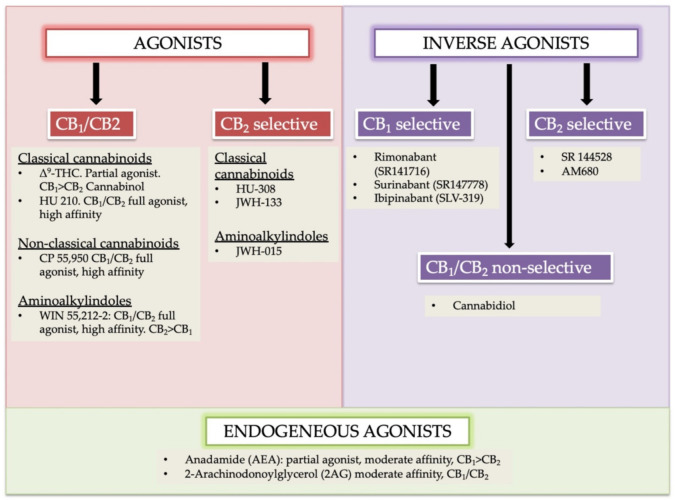
Agonists, antagonists, and endogenous agonist cannabinoids and their sub-types. Endogenous agonists: AEA and 2-AG; Exogenous agonist Δ9-tetrahydrocannabinol (Δ^9^-THC). The key psychoactive cannabis module, synthetic derivatives, HU 210, HU 308, CP 55,940 are identified. These compounds are commonly used as pharmacologic tools. Modified and reprinted from Ref. [24] with permission from Elsevier. License Number: 5066110366934. Abbreviation: CB_1_ (cannabinoid receptor 1); CB_2_ (cannabinoid receptor 2); HU-210, (highly potent cannabinoid receptor agonist); JWH-015 (a selective CB2 agonist); JWH-133 (a potent selective CB2 agonist); SR141716 (Rimonabant, a selective CB1 receptor antagonist or an inverse agonist); SR141716 (Rimonabant, a selective CB1 receptor antagonist or an inverse agonist); HU-308 (cannabidiol (CBD)-derivative drug); SLV 319 (a potent and selective CB1 receptor antagonist); CP 55,950 (a synthetic cannabinoid).

Cannabinoids receptors can be classified into four groups based on different chemical structures named as (i) classical (ii) non-classical (iii) aminoalkylindole (AAIs), and (iv) eicosanoid compounds. These groups are mostly heterogeneous [16,17]. The phytocannabinoids (Δ^9^-THC, Δ^8^-THC, cannabinol) and associated analogs (i.e., synthetic) form the classical group. *O*-arachidonoylethanolamine (virodhamine), arachidonoylethanolamide (anandamide), 2-Arachidonoyl Glycerol (2-AG), and other anandamide associated synthetic analogs/derivatives are present in the eicosanoid group. A cellular system of an organism usually produces the majority of ECs found in the eicosanoid group. Non-classical and AAIs groups contain synthetic cannabinoids. Both CB_1_ and CB_2_ receptors are the chief receptors of the ECS, and each of them possesses a different affinity of endocannabinoid agonists. For instance, the CB_1_ receptor has a greater affinity of Δ^9^-THC, and without selective marking of CB_1_/CB_2_ receptor(s), the phytocannabinoid cannabinol possesses partial affinity (Figure 1) [24].

### 2.2. Cannabinoids Receptors CB_1_ and CB_2_ and Functional Pathway

Recognized CBR CB_1_ and CB_2_ belong to the structural membrane receptors and family of G protein-coupled receptors. They also have seven transmembrane spanning domains. Limiting cellular response towards the specific cannabinoid receptor ligands, the effect of partial agonism is variable from its binding, and thereby inverse agonism to its functional selectivity plays a crucial role [25]. The functional influence of both CB_1_ and CB_2_ receptors is acquired when heterotrimeric G_i/o_ (a subunit of G protein) proteins are coupled. However, the activated CB_1_ receptors perform their functions due to G alpha i/o activation [26]. The inhibition of the adenylate cyclase enzyme synthesis is due to the attachment of CB_1_ to its agonist. The binding of CB_1_ to its ligand decreases the levels of cAMP and the elevated level of mitogen-activated protein kinase (MAPKs). Moreover, in few cases, the activated CB_1_ receptor corresponding to G_s_ proteins may accelerate adenylate cyclase cAMP [25,26,27,28].

**Figure 2:**
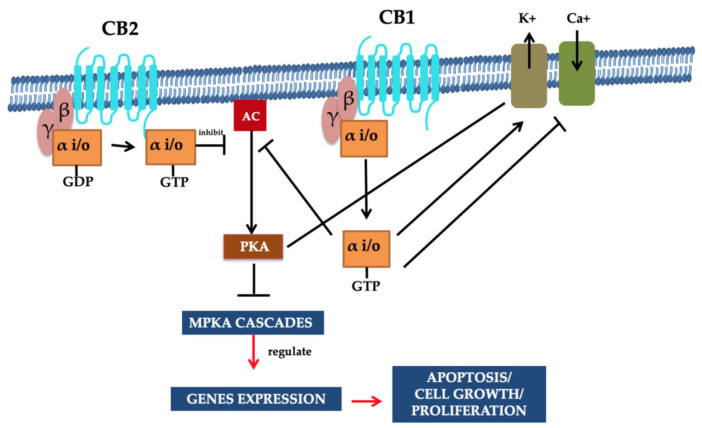
Signaling pathways of CB_1_ and CB_2_ receptors. G proteins are associated with CBR (i.e., CB_1_ and CB_2_). The inhibition of adenylyl cyclase activity and the stimulation of the variant MAPK cascades were demonstrated through these receptors’ activation. Moreover, the CB_1_ cannabinoid receptor facilitates the regulation of the voltage gated Ca^2+^ channels as they are negatively regulated, and inwardly resolving K^+^ channels are positively regulated. Intracellular free Ca^2+^ increase is prompted through phospholipase C (PLC) activation. The inhibition of gene expression is facilitated by the PKA, and reduction in cAMP directs cannabinoid-mediated inhibition. It is resulting in the MAPK cascade activation associated with cell survival or apoptosis. Such signaling pathways/mechanisms are associated with the multiple functions of the cells that are regulated through CBR. Reprinted from Ref. [24] with permission from Elsevier. License Number: 5066110366934.

In the cell membrane, both receptors (i.e., CB_1_ and CB_2_) and various ion channels, such as the potassium and calcium channels, are influenced positively because of independent activity. These ionic channels are activated when the cAMP-dependent interaction takes place between the molecules and the receptor. These molecules are recognized as protein kinase C, protein kinase A (PKA), p38, Raf-1, extracellular regulated kinase (ERK), N-terminal kinase (JNK), and c-fos, c-Jun [27,28]. The CB_1_ activation causes the lowering of the Ca^2+^ ions entry in the cell, which is the key factor for releasing neurotransmitters without cAMP association and results in reduced secretions of neurotransmitters. Therefore, a pre-synaptic receptor (CB_1_ receptor), when activated in a dose-dependent manner, leads to neurotransmitter release [29,30,31]. The regulation of the phosphorylation process and activation of various entities of the family of MAPKs, MAP kinase p38, and c-Jun can be performed through using both receptors. Consequently, JNK MAPK directs cell adhesion, proliferation, motility, and apoptosis. Glucose metabolism is also linked to gene expression [30,32,33,34]. Both receptors CB_1_ and CB_2_ that also respond via the stimulation of their agonists (exogenous/endogenous/synthetic). Agonist molecules are instantly deactivated when transported/uptake into cells, followed by their release and metabolic function. Both anandamide and 2-AG perform metabolism due to their enzymatic hydrolysis characteristics, and this process is carried out in combined activity of fatty acid amide hydrolase enzyme (FAAH) [35,36]. Furthermore, additional metabolic activities require monoglyceride lipase for the hydrolysis of 2-AG [37,38]. Figure 2 illustrates the summary of potential mechanisms of action [24].

**Figure 3:**
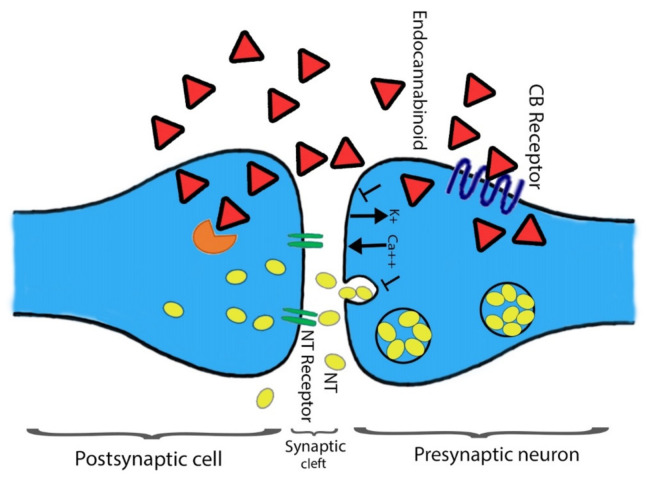
The action(s) of cannabinoids at the site of the presynaptic terminal. Agonists of a cannabinoid such as THC, 2-AG, and AEA attach to the CB_1_ receptor. The attachment is prompting alteration in intracellular levels of Ca^2+^ and K^+^ ions. Consequently, direct towards secretion of neurotransmitter blockage at the site of presynaptic neurons. At the postsynaptic neuron site, cannabinoids are devastated through the FAAH enzyme, and the respective metabolites are reused. Shapes representation: Red triangle for endocannabinoid; yellow oval spots for neurotransmitters; Orange color shape represents FAAH.

In addition to a few non-neuronal cells, central and peripheral neurons defined CB_1_ receptors [8,39]. The heterogeneous distribution of CB_1_ receptors is found among the CNS that aid the functional activities. Mainly all the functional activities controlling parts of the brain such as the cerebral cortex, entopeduncular nucleus, substantia nigrapars reticulate, hippocampus, caudate putamen, globuspallidus, cerebellum, and other areas of the brain and spinal cord possess the dense distribution CB_1_ receptors. The presence of CB_1_ receptors helps in processing or controlling the nociceptive information. Agonists’ ability associated with the CB_1_ receptor could be categorized using the distribution pattern of CB_1_ receptor among the CNS to impair cognition memory. Moreover, their potential role in changing the motor function and the development of an anti-nociception are also studied [40,41,42,43,44]. The terminal end of the central and peripheral nerves contains few CB_1_ receptors and is involved in controlling the release of excitatory and inhibitory neurotransmitters [8,45]. Primarily CB_2_ receptors found on immune cells are well-characterized and play a key role in immunomodulation [37,46,47]. Chemical messengers releasing capability is shared between CB_1_ and CB_2_ receptors. Initially, at the CNS, cannabinoids act with multiple neurotransmitters and participate in the modulation of their release through the function of the CB_1_ receptor [48,49] (Figure 3). Secondly, inflammatory cytokines release, and the immune system regulation require the prospective activity of CB_2_ receptors. In the following section, we have highlighted other types of CBR belonging to the ECS.

## 3. The ECS Associated Other CBR

### 3.1. Vanilloid Receptors

The cannabinoids receptors—except for CB_1_/CB_2,_ such as transient receptor potential cation channel subfamily V member 1 (TRVP1)—can have the ability to bind with cannabinoids receptors. TRVP1 is also known as the capsaicin receptor. In tissues (e.g., skin, blood vessels, heart, lung) and sensory neurons, it exists as a non-selective cation channel. In the peripheral nervous system associated with nociceptive neurons, mainly TRPV1 receptors are available; however, other CNS tissues also have TRPV1 receptors. Primarily, TRPV1 in afferent sensory and perivascular neurons is involved in transmitting and modulating pain (nociception) [50,51]. Moreover, the activation of afferent and perivascular neurons, and the TRPV1 activation using the endogenous cannabinoid receptor ligand anandamide facilitates the release of calcitonin gene-related and Substance P. Adverse effects such as pro-inflammatory, allogenic, and local vasodilatory were observed, although some advantages are also associated, such as anti-hypertensive and cardio protection [52,53].

### 3.2. Other Receptors

Various experimental studies verified that biotic factors for different cannabinoids are not directly associated with CB_1_/CB_2_ antagonists. In cannabinoid signal transduction receptor pathways, G-protein receptor 55 (GPR55) along with nicotine, the peroxisome proliferator-activated receptors (PPARs), 5-HT3, and adenosine A2A receptors are involved [21,54,55]. Experimental evidence presents the availability of allosteric sites on non-CBR apart from other receptors, (many but not all) for the binding of anandamide and some cannabinoids [8,56], like 5˗HT2 receptors [57], 5˗HT3 receptors [57,58], M1 and M4 muscarinic receptors, α1-adrenoceptors [59], as well as α˗amino˗3˗hydroxy˗5˗methy˗4˗isoxazolepropionic acid, glutamate receptors (i.e., GLUA1 and GLUA3) [60]. Exploring the potential significance of allosteric sites (partially or fully) on the M1 and M4 receptors or 5˗HT2 receptors requires more experimental research approaches [61,62].

## 4. Role of STAT Protein and Cannabinoids

In in vivo and in vitro experimental models, cannabinoids have been utilized in different anti-inflammatory activities and play an important role in diseases related to inflammatory degeneration, although the exact mechanism of action still requires more investigations [63] for the signaling pathways of cannabinoids involved in the anti-inflammatory functions. Studies illustrated the effect of various genes expression that is thought to be involved in inflammation reactions. Therefore, an induced inflammatory response is determined using the lipopolysaccharide (LPS) and microglial cell line of BV-2 mouse [64,65,66]. Consequently, along with marijuana, two major cannabinoids exist, THC and cannabidiol (CBD). These cannabinoids inhibit the synthesis and release of pro-inflammatory cytokines from LPS-activated microglial cells comprising interleukin-6, interleukin-1β, and interferon β (IFNβ) [67]. CBR (i.e., CB_1_ and CB_2_) or the abn-CBD-sensitive receptors do not actively participate in anti-inflammatory reactions. Moreover, the THC and CBD both act, using partially overlapping mechanisms [64,68].

NF-kB pathway is important to facilitate the regulation of the pro-inflammatory gene expression. The up-regulation of the NF-kB pathway supports the activation of the STAT3 transcription factor (Signal Transducer and Activator of Transcription 3), an entity of homeostatic action that induces anti-inflammatory events [5,69]. For treatment, CBD involvement with THC (partially) resulted in a lower level of transcription of mRNA for the Socs3 gene. Socs3 gene is recognized as the down-regulator of STATs, particularly for STAT3 [70]. While activation of the LPS-induced STAT1 transcription factor is reduced using THC and CBD. The LPS-induced STAT1 transcription factor down-regulation is the key element of IFNβ-dependent pro-inflammatory action [70]. Both THC and CBD possess diverse influences in pathways of the anti-inflammatory mechanism of action (e.g., IFNβ-dependent and NF-kB pathways) [69,71,72].

## 5. Immune System and The Role of Cannabinoids

Both CB_1_ and CB_2_ receptors are expressed in immune cells that are involved in the production of cannabinoids along with transports, release, and catabolic characteristics [21,73]. These immune cells include CD4 and CD8 leucocytes, B cells, NK (natural killer cells), monocytes, and neutrophils [74,75]. The range of expression of CBR depends on the state of cell activation and the stimulation and response of immune cells [73,76]. In an experimental mice model, the deficiency of FAAH increased the level of the anandamide in the CNS and periphery area [77]. Physiological involvement of ECs resulted in elevated levels of anandamide that mitigate the inflammatory activities and helps in reducing the immune system actions [73,78]. The results figured out that the ECS may potentially modulate the immune effects and proposed a theory that the exogenous cannabinoid involvement could have immunosuppressive effects. Therefore, it can lead to novel therapy development for diseases associated with inflammation and autoimmunity. Later on, many studies underpin the theory of this association [73,79].

Furthermore, Δ^9^-THC can support the modulation of the Th1/Th2 balance by using the controlled immune actions of Th1 and the increasing level of Th2 [73,80]. However, Δ^9^-THC as a therapy recommendation is limited because of its psychoactive effects. Alternatively, cannabidiol possesses less affinity for both CB_1_ and CB_2_, resulting in null psychoactive effects [8,81,82]. Furthermore, some studies also proposed that the frequent administration of cannabidiol to humans does not have adverse side effects [83,84]. Primarily cannabinoids pretend their immunosuppressive features in four pathways: (1) apoptosis induction; (2) cell proliferation inhibition; (3) cytokines inhibition, and (4) production and induction of chemokine associated with regulatory T cells [85,86,87]. Among mitogen-induced human T lymphocyte and B lymphocyte (human neuroblastoma cells (CHP-100) and U937 lymphoma cell lines, respectively), anandamide induces dose-dependent apoptosis [88,89]. While Δ^9^-THC can trigger the apoptosis cycle (programmed cell death) in murine macrophages and T cells via regulating the activity of BCI-2 (B cell lymphoma-2), and also this cycle triggers by the action of caspases (cysteine-dependent aspartate-directed proteases, cysteine-aspartic proteases, cysteine aspartates) [90]. Because it is tough to detect the in vivo rate of apoptosis—as apoptotic cells are rapidly cleared through the pathway of phagocytes [91,92]—the induction of apoptosis by engaging the cannabinoids cannot be confirmed in in vivo results.

Non-activated lymphocytes are significantly affected by Δ^9^-THC as compared to activated lymphocytes. The cell line treated with THC exhibited more significant apoptosis levels compared to the cell line treated with both Δ^9^-THC and mitogen [92,93]. The expression of CB_2_ is down-regulated by the activated lymphocytes, which decreased the sensitivity towards Δ^9^-THC. In lymphocytes, induced thymocytes apoptosis is inhibited through Δ^9^-THC via administrating the CB_2_ antagonists. However, CB_1_ antagonists showed non-significant effects. Therefore, Δ^9^-THC performs function through CB_2_ receptors to induce apoptosis [94]. In a dose-dependent approach, cannabidiol induces apoptosis on CD^4+^ and CD^8+^ T cells. Cannabidiol facilitates the production of reactive oxygen species (ROS), and the activation of caspase-8 and caspase-3 during apoptosis [95]. In vitro studies in both humans and mice showed that the increased and decreased concentrations of Δ^9^-THC stimulate the T cells and inhibit responses to the LPS, anti-CD3 antibody, and T cell mitogens [96]. A study demonstrates the two-phase action of cannabinoids on B cells: Firstly, increased proliferation of the B cell in response to Δ^9^-THC, and secondly, inhibited B cells response to LPS after the administration of cannabinoid [96,97]. In other experimental models, Δ^9^-THC played a role in suppressing immune functions and increased the chances of infections. The exact pathway which is adopted to suppress the immunity by Δ^9^-THC is not yet fully understood. The earlier study illustrates that the induced inhibition of lymphocyte proliferation can be directly achieved on the immune cells instead of CB_1_ and CB_2_ receptors modulation. However, this activity was detected in the subsistence of antagonists of CB_1_ and CB_2_ [91]. The proliferation of thymocytes restrained through Δ^9^-THC because of the inhibition of calcium stabilization within the cell [98,99]. The inhibited proliferation of T lymphocyte and B lymphocyte was achieved in response to mitogen stimulation, and decreased the concentration of anandamide [100,101].

Cannabidiol inhibits the interleukin (IL1, IL12), tumor necrosis factor (TNF)-α interferon-gamma (INF-γ) cytokine secretion, and also the synthesis of Th2-associated cytokines through peripheral mononuclear blood cells [95]. The activity of prostaglandin E2 and tissue cyclooxygenase enzymes (COX) is restrained due to cannabidiol action. Low efficacy of Δ^9^-THC and cannabidiol supports the modification of TH1 immunity to protect TH2 immunity [91,96]. Cannabinoids may play a vital role in monocyte differentiation to macrophage phenotypes M1 or M2 and their capability to synthesize chemokines, cytokines, and the regulation of different mediators of the immune system [102,103,104].

Different people who commonly take cannabis are not responding differently to cannabinoids, particularly within the immune system. In exposed entities (non-naïve) to cannabis, a more than twice-a-week migration of monocyte may be inhibited via phytocannabinoids. In contrast, the entities (naïve) do not affect if not exposed in the last three years [105]. In a study, both ECs and synthetic cannabinoids had no adverse effects, and researchers demonstrated the association between these two. Cannabis (non-naïve entities) expressed a four-fold increased concentration of mRNA of CB_1_ receptor than naïve entities of cannabis, although the mRNA of the CB_2_ receptor remained the same. Therefore, the sensitivity of isolated monocytes from non-naïve entities correlates to the increased expression of CB_1_ receptors [8]. In *Legionella pneumophila* (Lp) infected dendritic cells, Δ^9^-THC possessed immunosuppressive effects. Immunization potential was restrained in Lp loaded cells in the pretreated (with Δ^9^-THC) dendritic cells in mice. In a ConA induced hepatitis model, liver damage is increased due to Δ^9^-THC, elevating levels of apoptosis within activated T cells, and the induction of T reg cells to inhibit the induced cytokines in vivo [87]. It is recommended that T reg cells can resist inducing the apoptosis via Δ^9^-THC and control the T cell activation which escapes from apoptosis [87,106]. Cannabis, particularly cannabinoid receptor ligands, help in the suppression of serum immunoglobulin (Ig) levels [107,108]. Decreased B cell count was identified via Δ^9^-THC, including IgG, and IgM levels were also reduced [109]. There is a difference in opinion that exists about the level of B cell [110]. In mouse peritoneal macrophages, the spreading, phagocytosis, cytosis, the production of cytokine, and presentation of the antigen are suppressed through cannabinoid receptor ligands in vivo. However, in vitro studies showed the suppressed actions NK cells and cytotoxic effector [111].

Despite the entire discussion, few studies recommend that cannabinoids might have pro-inflammatory effects [112,113]: allergic responses [114]; CB_1_ receptors secreted mast cells inflammatory mediators [113], as well as the elevated proliferation of B cells [115]. Understanding the potential role of cannabinoids in the immunomodulation function is key. The subsequent factors are dependent on the types of cannabinoids, cell type, and the administrated concentration. Within immune cells, the optimal dose of cannabinoids leads to induced apoptosis that enhances the inflammatory actions and shields the host from acute and chronic inflammation responses. Therefore, they can be beneficial if the suppression of immune response is required. Additional clinical research is required to validate such studies in human entities [116,117,118].

## 6. Autoimmune Disorders and Cannabinoids

### 6.1. Multiple Sclerosis (MS) and Cannabinoids

Multiple sclerosis is recognized as a human CNS chronic neuro-inflammatory autoimmune demyelination disease. The T lymphocytes enable multiple sclerosis. The inflammatory process responds when the disruption of BBB leads the cause of inflammation, macrophages activation, and synthesis of cytokines and “cytotoxic” proteins like metalloproteinases. In an experimental mice model of multiple sclerosis (EAE (experimental autoimmune encephalomyelitis)), it was reported that CB_1_ receptors perform a primary function in administering neuro-inflammation. Mice deficient in a CB_1_ receptor sustain inflammation, and excitotoxic effects create substantial neuro-degeneration, consequently influencing the immune system [119,120]. In an EAE model, neuro-degeneration inhibition has been regulated by mediating THC. Thus, the reduction of inflammatory cytokines within cerebrospinal fluid and the associated level of glutamate was observed [121]. In the cerebral cortex, glutamate is the primary excitatory neurotransmitter. When it releases at increased levels, it can be associated with neurodegenerative disease(s). In the CB_1_ receptor knockout mice studies, increased susceptibility to neuro-filament damage, caspase3 activation, and huge neuro-filament H epitope dephosphorylation during chronic replacing EAE were considered markers of axonal injury. The signaling pathway associated with the CB_1_ receptor suggests neuroprotection using the EAE involvement [122].

The CB_1_ receptor mediates down regulatory responses in the CNS. Human tested models administered with CB1 agonists showed improvement in motor disorders such as spasticity, tremor, and ataxia [119,120]. Rolipram is a well-recognized phosphodiesterase-4 inhibitor developed in the last decade of the 20th century as a potential antidepressant. Different types of rolipram inhibit EAE to reduce the clinical ineffectiveness of reduced motor inhibition. Basal ganglia normalization in EAE rats is also associated with the CB_1_ gene expression [123,124]. In EAE rats, endocannabinoid concentration was categorized based on levels of anandamide and 2-AG in motor-linked areas of the brain. Mainly, motor-linked regions of the brain include the striatum, midbrain, and other brain regions [125]. CB_2_-selective agonists (i.e., RWJ400065) do not exhibit the inhibition of spasticity. During EAE in the CNS, CB_2_-deficient T lymphocytes perform restricted levels of apoptosis, elevate rated proliferation, and massive synthesis of inflammatory cytokines that result in lethal clinical disease(s). Furthermore, CB_1_, CB_2_, and FAAH selective glial expression have been categorized in association with multiple sclerosis. In the pathogenesis of the current disease, these components have supporting roles for the ECS [126].

During endogenous cannabinoid MS progression, anandamide performs an essential role in administrating the inflammation process. Selective anandamide treatment using an inhibitor (UCM707) demonstrated that there is a minor progression in motor function and reduced microglial activation during established disease. UCM707 can reduce the expression of MHC class II antigen, decrease the cellular infiltrates, and synthesize proinflammatory cytokines (i.e., TNF-α, IL-1β, IL-6) in the spinal cord. In CNS inflammation conditions, the highly activated ECS protects neurons by anandamide from inflammatory damage via the CB_1_/CB_2_-mediated rapid induction of mkp-1 microglial (mitogen-activated protein kinase phosphate-1) [127]. Although infrequent data is available in human models, a clinical trial resulted in the effect of Δ^9^-THC being administered in a group of patients (i.e., ~498) along with initial or secondary stage multiple sclerosis. There were no significant variations recorded in all associated trials of the progression of MS. However, the clinical variation might have affected the potential progression rate lower than anticipated [126,128,129,130].

### 6.2. Cannabinoids in Rheumatoid Arthritis

Inflammatory and degenerative joint diseases are pathologically characterized or associated with the loss of articular cartilage. There is an elevated cartilage breakdown in osteoarthritis and RA. This is induced by adopting the progressive synthesis of inflammatory cytokines, particularly IL-1, and synthesis of TNF through the synovium cells or articular chondrocytes [131,132,133]. Previous studies suggested an increase in specific matrix metalloproteinases (MMP-3 and MMP-13) are responsible for destroying cartilage. In animal models of arthritis, cannabinoids demonstrated anti-inflammatory effects and reduced damage of the joint [134,135]. In vitro studies proposed that cannabinoids restrain the synthesis of a cytokine through RA fibroblast. Furthermore, matrix metalloproteinases (MMPs) are secreted from fibroblasts as synovial cells [131,136]. Interleukin 1(IL-1) reduction leads to the induction of proteoglycan and degradation of collagen within bovine cartilage that consequently limits the breakdown of extracellular cartilage matrix mediated by cannabinoids [137].

Synthetic cannabinoid(s) [i.e., WIN-55,212-2] are agonists of both receptors, such as CB_1_ and CB_2,_ and these can activate different receptors, including proliferator-activated alpha and gamma (PPAR α and γ) receptors of peroxisome [138,139,140]. The MMP-3 and MMP-13 protein(s) gene expression can be reduced using the synthetic cannabinoid WIN-55,212-2 during IL-1b availability. It signifies that cannabinoid might perform better function(s) in the case of arthritis therapy. Moreover, gene expression of tissue inhibitors of matrix metalloproteinases (i.e., TIMP-1 and TIMP-2) substantially reduces it to below basal levels [141,142]. In vitro studies were organized to check the efficacy of CB_2_R agonist [HU-308 (Hebrew University-308), an agonist for peripheral cannabinoid receptor CB_2_] and IL-1β-induced proliferation (e.g., fibroblast-like synoviocytes) was inhibited in pretreated cultured RA fibroblast with an agonist HU-308.

The synthesis of pro-inflammatory cytokines (particularly IL-6) and matrix metalloproteinases (MMP3 and MMP13) are decreased by the associated agonist and were linked with cartilage destruction. Another report represented that non-psychoactive cannabinoid ajulemic acid (AJA) reduced the secretion of matrix metalloproteinases (i.e., MMP-1, MMP-3, and MMP-9) from fibroblast. Therefore, IL-1a plus TNF-α activated synovial cells [143,144]. An in vivo work reported that AJA expression responds to the declining level of Adjuvant-induced arthritis [145]. In the mouse model for arthritis study, the synthesis of TNF mouse macrophages was inhibited in response to the administration of a new synthetic cannabinoid HU-320 (Hebrew University-320). The researcher observed significantly reduced reactive oxygen intermediates and a rise in the level of serum TNF suppression [141,142]. These experimental studies recommended the potential anti-inflammatory characteristics of cannabinoids and proposed their use to treat inflammatory arthritis. It is confirmed that the use of cannabinoids in human RA reduced inflammation. However, other reports pretend the role of cannabinoids in the therapy of pain was associated with RA. A significant primary role of the ECS is performed in the peripheral regulation of nociception [143,144]. Nociceptor terminals are available on CB_1_R that may exhibit anti-nociceptive and anti-inflammatory functions of contiguous synthesized *N*-arachidonoyl ethanolamine. In addition, they performed the inhibitory effect(s) on the secretion of excitatory neuropeptides [8,146].

CB_1_R and CB_2_R are distributed in the dorsal root ganglia, and their activation reduces the transmission of nociceptive [52,147]. The ECS administers nociception that is controlled by the CB_1_R available at the supraspinal and spinal stage [52,148]. According to pharmacological studies, peripheral endocannabinoid basal activity recommended the expression of nociceptor activity in osteoarthritic joints [149]. In synovial biopsies started from total knee arthroplasty of patients suffering from advanced RA and osteoarthritis, both mRNA and protein were presently associated with CB_1_R and CB_2_R [150]. In the synovial fluid of patients suffering from this disease, both anandamide (AEA) and 2-arachidonoylglycerol (2-AG) were identified, but no solid evidence was about the operational mechanism of the ECS in osteoarthritic joints [150]. In the rat model-based osteoarthritis studies, the level of spinal cord AEA, 2-AG, and their synthesizing enzymes were higher [151,152]. Briefly, these studies provide some preliminary basis regarding the involvement of the ECS during osteoarthritis disorder. However, few experiments were conducted using a human experimental model, and the available data does not provide smooth information because there are disagreements between different research groups. Very few research data are available about rheumatic diseases that show the effectiveness of cannabinoid nabiximol on chronic pain.

### 6.3. Cannabinoids in Scleroderma

In scleroderma fibroblasts, the overexpression of both CBR (i.e., CB_1_ and CB_2_) was carried out to compare with healthy and normal fibroblasts. The effect of agonist (WIN55,212-2) of both receptors was observed in the synthesis of the collagen. In the skin fibrosis study, inhibition of type-1 collagen and procollagen type-I PIP supernatant levels were detected. Supernatant levels of both types were directly linked with the changing concentration of both agonists (WIN55,212-2) [153,154,155]. The decrease in the growth factor contributed to the fibrosis process, TGF-β and CTGF (a downstream mediator of TGF-β) adversely affected the synthesis of type-1 collagen. Similar results were acquired in scleroderma pathogenesis that involved the fibroblast transdifferentiation inhibition and over-expression of IL-6 in scleroderma cultures [32,156].

The agonist WIN55,212-2 raised a significant count of the scleroderma fibroblasts during apoptosis, and these results were found during the anti-proliferative effect of cannabinoids [157]. As a result, the toxic effect of WIN55,212-2 was not observed in cell cultures. However, the “classical” both CBR (i.e., CB_1_ and CB_2_) did not possess the anti-fibrogenic effect of WIN55,212-2 [158]. Further studies showed that signal transduction pathways of CBR may additionally consist of: (a), transient receptor potential vanilloid type-1 (TRPV1); (b), adenosine A2A receptors; (c), GPR55 and nicotine; (d), 5-HT3; (e), the peroxisome proliferator-activated receptors (PPARs) [21,157,159]. Oral administration of non-psychoactive synthetic analog/derivatives of tetra-hydrocannabinol (ajulemic acid (AjA)) has a binding affinity with PPAR-γ (peroxisome proliferator-activated receptor-γ). This attachment inhibits the skin fibrosis development and decreases the thickness of skin approximately to the control stage. At the basic biological stage, a dose-dependent reduction of procollagen and 5TGF-β was utilized [157,160]. It is also hypothesized that the stimulation of PPAR-γ might possess an anti-fibrotic effect.

### 6.4. Type-1 Diabetes and the Role of Cannabis

Pancreatic β-cells in type-1 diabetes mellitus are prone to damage through lymphocytes CD4Th1 and CD8T [161,162], including the preliminary laceration of this syndrome, leukocytes, specifically lymphocytes and the islets are infiltrated during insulitis type-1 diabetes mellitus [163]. The presence of diabetes was significantly controlled from 86% to 30% (in non-treated mice vs. treated mice with cannabidiol). The experimental study was conducted for six to twelve-week-old non-obese-diabetes (NOD), and cannabidiol treatment significantly reduced the plasma levels of INF- γ, TNF-α, and other TH1 cytokines in experimental mice. Concurrently, the synthesis of IL4 and IL10 (TH2 cytokines) was increased. Histopathology investigations of pancreatic islets extracted from mice treated with cannabidiol revealed the subsequently reserved insulitis. However, key insulitis and associated TH1 cytokine can be delayed or inhibited using cannabinoids in the NOD mice model [64,161].

Cannabinoid might inhibit diabetes type-1 in NOD young mice (i.e., ~four-week-old). Later on, similar research was carried with eleven to fourteen weeks old NOD mice treated with cannabidiol. The occurrence of diabetes was increased at the end of the treatment from 86% to 100% in control non-treated mice. However, treated mice with cannabidiol only showed 32%. Histological analysis revealed that cannabidiol treated mice pancreas exhibits more intact islets than in the control group mice [64,161]. Thus, Cannabidiol might have a potential primary function in the prevention mechanism of human type-1 diabetes. However, the mechanism of auto-immune characteristics of this molecule requires further investigation. It is important to study the deviation response of Th1 if it possesses the non-specific characteristics as an immunosuppressive agent. Cannabidiol is initiating the aberration when Th1 transforms into Th2. It is hypothesized that it might be recruited to prevent diabetes before the complete β-cells destruction in early on-set patients and maybe in high-risk patients. Before the destructive response of Th1, the auto-immune response is initiated to protect the response of Th2. Therefore, cannabidiol-based treatment is no longer needed, excluding the concern to more extended protection effects [64,161]. Alternatively, if there is a mechanism of non-specific immunosuppression, then it requires associated permanent therapy. Critical potential risks are linked with the extended therapy options, such as enhanced vulnerability to devious dangerous infections and the possibility of enhanced malignancy. Some risks are directly connected to diabetes; however, they can be controlled along with intensive therapy of insulin recommended based on glucose levels [64,161]. The type of therapy for other linked risks is essential to be carefully evaluated before applying human cannabinoid therapy.

The immune response of either single or both Th1 or Th2, cytokines mediates a critical immunomodulatory role, and macrophages secrete cytokines [164]. In the macrophage-depleted NOD mice model, the IL-12 administration is recognized as a potential cause of the disease [64]. However, the synthesis of IL-2 macrophage was significantly reduced by the cannabidiol [165]. The reduction of IL-2 triggers the TH2 discrepancy along with reduced INF-g synthesis. Enhanced IL-12 effects stimulated the cytotoxic T cells, synthesis of TNF-α by macrophages, IL-1, IL-6, and nitrogen-free radicals by INF-γ. These molecules are linked with the destruction of β-cells [64,161]. Subsequently, macrophage IL-2 synthesis might involve suppressing Th1-mediated auto-immunity [78,166]. Further studies are required to confirm if essentially Th1/Th2 shift within humans gives long-term protection from disease.

### 6.5. Obesity and Endocannabinoids

ECs have a considerable effect on the body mass index and take part in regulating glucose metabolism by mediating the lipophilic molecules [167,168]. The clinical trials recommended that peripheral CB_1_ receptors mediated glucose metabolism regulation and suggested that adiponectin levels, lipids, and glucose metabolism are affected when the CB_1_ receptor is blocked. As a result, the weight is lost [167,169,170]. The signaling pathway and synthesis of endocannabinoid(s) are commonly dependent on externally stimulated factors such as metabolic dysregulation, cellular stress, and damage of tissue(s) [169,171]. Although ECS regulation is directed to change in response to obesity, the peripheral organs or the brain region of the body in response to obesity or after a high-fat diet stimulate the ECS [146,169,172]. A potential mechanism of the activation of the ECS may consist of an increased level of fatty acids in the diet. This might be a possible pathway/mechanism; however, the FAAH reduced the degradation of enzymes in the liver in diet-induced obese mice [167]. Recently, it reported that obese post-menopausal women described the increased level of ECs, down-regulation of FAAH gene expression, and subcutaneous adipose CB_1_ [169]. Bluher et al. explained the significant changes in ECS regulation during obesity and highlighted the increased level of circulating ECs. The increased level of ECs is directly correlated to the visceral adipose tissues, and abdominal obesity associated with the ECS can treat metabolic changes as a primary target [173].

### 6.6. Cannabis and Inflammatory Bowel Diseases

**Figure 4:**
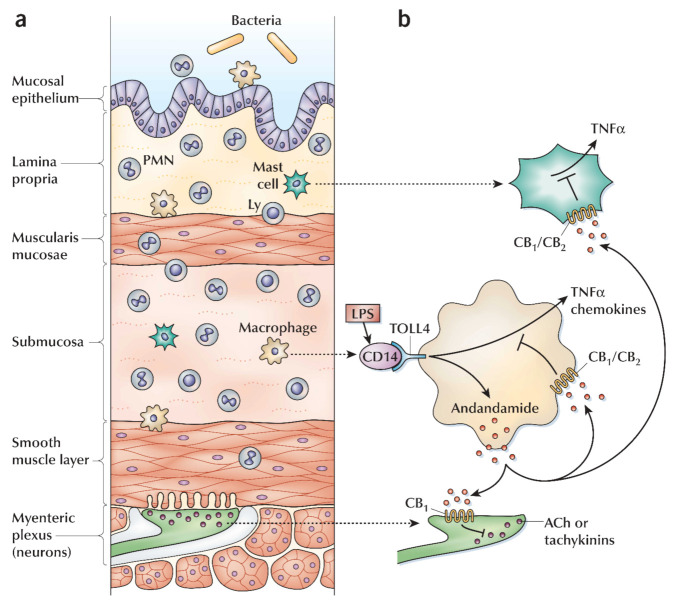
Anti-inflammatory ECs recommended targets and cell types in inflammatory bowel disease. (**a**), Inflamed bowel cross-section along with infiltration of the leukocytes (i.e., lymphocytes (Ly), polymorphonuclear leukocytes (PNM), macrophages, and mast cells). (**b**) In macrophages, the synthesis of the MIP-2 macrophage inflammatory protein-2 and CXCL-8 (chemokines), TNF-α (Tumour necrosis factor α), and anandamide initiated by LPS. Anandamide performs autocrine mediation to inhibit the synthesis of TNF-α and synthesis of the chemokine. The inhibition is carried out using receptors CB_1_ or CB_2_ (one of two or both). The activation of both receptors CB_1_ and CB_2_ can inhibit the synthesis pathway of TNF-α in mast cells. Consequently, inflammation reduction and leukocyte infiltration processes are affected. Stimulation of CB_1_ receptor paracrine on extrinsic and intrinsic enteric neurons blocks the release of acetylcholine (ACh) and tachykinin, respectively, that results in the inhibition of gut motility. The use of the FAAH inhibitor prevents the anandamide breakdown. Reprinted from Ref. [180] with permission from Springer Nature. License Number: 5066110580718. Abbreviation: CD14 (cluster of differentiation 14); TOLL4 (toll-like receptor 4 (TLR4)); LPS (lipopolysaccharide).

Cannabis is crucial for the treatment of bowel inflammation. However, actual mechanisms are not yet fully described; possibly CB_1_ and CB_2_ can take in mild and acute conditions [5,174]. In the last decade, several animal model-based studies have determined the probable effects of cannabinoids for possible therapy in colitis (inner lining inflammation or inflammatory reaction inside the colon) conditions. Numerous studies have confirmed that cannabinoid agonists act as regulators in inflammatory response via the cannabinoid receptor [174,175]. In humans, distinct expression of IBD CBR performs regulatory roles of cannabinoid in the disease condition [176,177,178,179]. In ulcerative colitis (UC), anandamide and producing enzymes are decreased, and the expression of the CB_2_ receptor and associated enzymes are increased for the production and degradation of the 2-arachidonoylglycerol [180,181]. The ECS is activated during gut inflammation, although inflammation may be reduced through endogenous anandamide [176,177,180,181,182] (Figure 4). Both receptors CB_1_ and CB_2_ are distributed at the colonic epithelium; therefore, a high level of cannabinoids might be involved in the wound closure in the colon epithelial [176,183]. The CB_2_ leads to apoptosis, and in colitis, it inhibits the proliferation of T cells and reduces the neutrophils utilization, followed by macrophages to the inflamed colon. In the enteric nervous system, the ECS may control the gut motility and the secretion(s) using the CB receptors [5,64]. During IBD, CB_1_ receptor(s) may protect the bowels from hyper-stimulation. This mechanism facilitates THC-based IBD symptoms, primarily disease diarrhea; furthermore, it involves inflammation/inflammatory reactions [5,64]. Patients suffering from UC specifically stated that cannabis stimulates the recovery pathway from diarrhea. In the CNS, stimulation of the CB_1_ receptor decreases pain sensation and nausea. Moreover, peripherally restricted CB_1_ and CB_2_ agonist was too weak to enhance colitis, and these facts strengthened the proposed theory [180].

A placebo-controlled cohort study was conducted among 21 patients suffering from Crohn’s disease [184]. In 50% of patients, cannabis-induced clinical remission and 80% of the total patients did not respond to anti-TNF-α, while in comparison to a placebo-controlled cohort study, only suggestive improvement occurred, with no induced remission. However, a declining trend was observed among participants when cannabis therapy was carried out. Another study conducted among patients with IBD (via cannabis) recorded that abdominal pain was enhanced [185]. Ref. [186] found that therapeutic cannabis was linked to the recovery process from Crohn’s disease. Some patients suffering from UC and CD use cannabis for appetite enhancement [187]. A significant rise in the individual(s) weight was noticed as a result of using (i.e., ingesting) cannabis [188]. It can be hypothesized that cannabinoid therapies could have useful effects on IBD, although more trials are needed to recognize effective cannabinoid types, mode of intake, and the recommended dose [186].

### 6.7. Thyroid Cancer

In the thyroid gland, thyroid carcinoma is the most antagonistic form of cancer. Anaplastic thyroid carcinoma cell line (ARO) consists of an IL-12 gene with an anti-tumorigenic effect [189,190]. The anti-tumorigenic impact was noticed in response to the activation of the cannabinoid receptor. Different researches agreed that apoptosis activity is induced in cell lines ARO and ARO/IL-12 and CB_2_ agonist JWH-133 and CB_1_/CB_2_ agonist WIN-55,212-2 both facilitate the apoptosis [189,191]. In carcinoma cells of the thyroid, 2-methyl-2’-F-anandamide (Met-F-AEA) induced apoptosis, and the apoptosis pathway is mediated through p53 and p21 [192,193].

### 6.8. Ovarian Cancer

**Figure 5:**
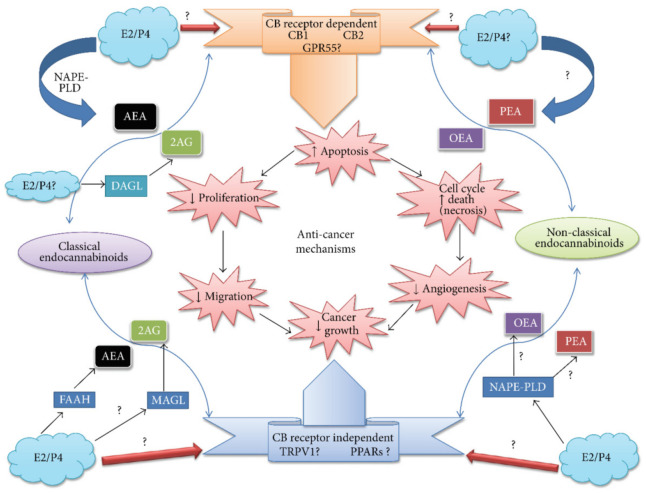
Putative collaborations between sex hormone-dependent cancers and the ECS (endocannabinoid system). The production of ECs is carried out by the actions of specific enzymes (light yellow boxes and broad blue arrows). These actions are supported by the enzymatic hydrolysis of NArPE lipid precursors, particularly associated with the membrane. Both sex steroid hormones perform theoretical roles in the pathway. Both hormones, such as progesterone (represented as P4 at each corner in the circle filled with light blue color) and oestradiol (represented as E2 at each corner in the circle filled with light blue color). 2-arachidonoylglycerol (2-AG) and anandamide (*N*-arachidonoylethanolamide, AEA) are known as classical ECs. The non-classical ECs are recognized as PEA and OEA. Both forms of ECs (i.e., classical and non-classical) bind independently or in concert at receptors CB_1_, CB_2,_ and GPR55. Receptors PPAR and TRPV1 (represented in gold cold fill boxes) involve in the anticancer mechanism. Green boxes show the important activities linked to the anticancer mechanism in the center of the figure. There are speculated observations; either all these activities are interconnected or perform functions independently. Although this mechanism significantly reduced the mass of cancer cells. The ECS and its adjusting boundaries with sex steroid hormones are represented in the anticancer mechanisms, though theoretical or unidentified associations are specified using a question mark(s). ↑ ↓ Arrows illustrated the increasing or decreasing activity of a particular anticancer mechanism. Reprinted from Ref. [196] with permission under open access article distributed under the Creative Commons Attribution License. Abbreviation: NAPE-PLD (N-acyl phosphatidylethanolamine phospholipase D); OEA (oleoylethanolamide); PEA (palmitoylethanolamide); PPAR (peroxisome proliferator-activated receptor); TRPV1 (transient receptor potential vanilloid type-1); AEA (arachidonoylethanolamide); FAAH (fatty acid amide hydrolyse); MAGL (monoacylglycerol lipase); DAGL (diacylglycerol lipase).

The ECS exists in CNS and peripheral areas of the body, and the study of sex steroid hormones validated these verdicts. The synthesis of progesterone steroid hormone C-21 occurred in the ovarian corpus luteum after ovulation that probably contributed to endocannabinoid signaling regulation. Human lymphocyte FAAH activity is upregulated by the expression of progesterone (via the transcription factor Ikaros) [194,195] that consequently reduced AEA levels in plasma [196]. This system controls various physiological roles and behavior of the cellular pathway, as presented in Figure 5 [196]. Moreover, in immortalized human lymphoma cells line (U937 cells), progesterone tends to increase the FAAH expression level and related activity. In contrast, the immortalized cell line of human neuroblastoma (CPH100 cells) did not possess these specific characteristics [196]. It has been reported to have an insignificant effect in lymphocytes on EMT (endocannabinoid membrane transporter), NAPE-PLD (N-acyl phosphatidylethanolamine phospholipase D), and CB_1_ expression [196]. The expression of uterine NAPE-PLD has been down-regulated in the mice model. The down-regulation of uterine NAPE-PLD modulates the reduction of AEA levels [197] in tissues and performs a function to down-regulate the FAAH activity in the uterus of a pregnant mouse [194,195]. However, when accounted for both together with a discreet expression of NAPE-PLD in mice, the FAAH ratio’s activity can be significant to regulate AEA levels in the mouse uterus and thereby maintenance of pregnancy or pathologies of endometrial (uterine cancer). In brief, the progesterone action on AEA levels in the uterus of rats is more complicated when progesterone activates its synthesis in the ovariectomized animal [194,195]. All studies mentioned the ovarian hormones affect the synthesis of anandamide. The change in the physiological conditions significantly affects the performance of ovarian hormones. These physiological conditions may be dependent on activated blastocyst, estrous cycle, and early pregnancy. The exact feedback mechanisms associated with all these activities are not yet fully known, although a sheep model of experimental studies illustrated progesterone level, corpus luteum weight, mRNA ration concerning corpus luteum LH receptor, and LH receptor density [194,195]. In pregnant rats, the progesterone levels in serum and LH content participate in the administration of AEA levels [195]. Therefore, the co-regulation of ECs and progesterone function may be linked with positive and negative feedback mechanisms.

The 17β-oestradiol (E2) is known as the most effective growth stimulator form of estrogen. The E2 has a direct and/or indirect association with the ECS. Such E2 association assisted NAPE-PLD activation and the blocked FAAH production. It triggers endothelial cells to secrete AEA [8,194,196]. In comparison, another study revealed that estradiol downregulated the NAPE-PLD within the uterus, consequently reducing anandamide levels; however, this is not inveterate yet [198]. Some studies elaborated restrained activities of FAAH via E2 in mouse uterus, despite the regulatory fact of FAAH expression [194,195]. Moreover, estrogens appear to perform the regulation mechanism of the ECS in other types of cancers [195]. In sex steroid hormone-dependent tumors, the discrepancy of estrogen effects on the ECS signaling pathway depicted a complicated interaction that performs a critical role. In the following paragraph, we emphasized providing details of endometrial cancer.

Endometrial cancer is the seventh most commonly and globally diagnosed malignancy [199], and the fourth most commonly diagnosed gynecological cancer identified in the UK in 2008 [200]. Endometrial cancer refers to numerous forms of malignancies. These malignancies evolve from the endometrium, obesity, early menarche, null parity, late menopause, and estrogen-only hormone replacement-based therapy. These factors are potentially documented as high-risk factors that lead to endometrial carcinoma. More exposure to estrogen plays a crucial role in the etiology of endometrial cancer [201,202]. Menstrual disorders, obesity, and endometrial cancer are directly linked to chronic inflammation [201,202]. Additionally, endometrial cancer risk can be enhanced due to endometriosis and uterine fibroids because such malfunctions are linked with excess concentration of estrogen and pelvic inflammation [201,202]. Endometrial cancers are of two types based on clinical and molecular characteristics. The first type is estrogen-dependent endometrioid carcinomas (EECs), or type-I, and is observed in approximately 80% of the cases. Type-1 expresses receptors linked to estrogen (ER) and progesterone (PR) biomolecules. Type-I is exhibited in premenopausal and postmenopausal young women [203,204] and strongly connected with unrestricted exposure, either exogenous or endogenous of estrogen. The second type is non-endometrioid endometrial carcinoma (NEECs) or type-II, consisting of distinct papillary serous and indistinct cell carcinomas [201]. In different types of endometrial cancers, the expression of endo-CBR (i.e., CB1 and CB2) has been specified. The expression of the CB2 receptor was identified using immunohistochemistry independently in the cells of endometrial cancer or endometrial tissues. CB_2_ protein expression was considerably increased in tissues of endometrial cancer as compared to healthy endometrial tissues. Moreover, the immunoblotting analysis showed that insignificant changes were prominent in CB_1_ expression.

Mass spectrometry findings indicated an insignificant increase in levels of AEA or PEA that suggested the 2-AG up-regulation in endometrial cancer tissues. Immunoblotting analysis showed the selective down-regulated expression of MAGL in endometrial cancer tissues as compared to normal tissues. However, there were minor differences in FAAH protein expression. The regulation mechanism of the CB_2_ receptor is changed in endometrial cancer because of a significant increase in the level of CB_2_ in the cell line of human endometrial carcinoma (AN_3_CA) compared to healthy cells. For the expression of endocannabinoid receptor CB_2_, both types of cells (i.e., AN_3_CA and healthy cells) were transfected with cDNA comprising plasmid [201,202]. Recent studies revealed the diversified endogenous pathway for CB_2_ in endometrial adenocarcinoma. In the etiology of endometrial cancer, the endogenous way for CB_2_ can be one of the fundamental factors primarily linked to the regulatory pathway of the ECS. In endometrial cancer tissues, the ultimate aspect for the growth of endometrial cancer is a significant increase in the expression of CB_2_ receptors and level of 2-AG. These changes resulted in a primary difference in the ratio of estrogen/progesterone; however, further studies are required to validate the observations. In AN_3_CA transfected cells, the effect of CB_2_ has been studied, and the mitochondrial function of the cell was reduced by 40% compared to healthy cells [205]. Such an effect could not enhance by using an agonist of the CB_2_ receptor, i.e., JWH133, although it is inhibited through the antagonist of the CB_2_ receptor, i.e., SR144528. A new diagnosis method for endometrial cancer can be the increased expression level of CB_2_ receptors, particularly in tumor-developing cells. Subsequently, specific CB_2_ agonists could facilitate the development of novel antitumor compounds to treat endometrial carcinoma because those compounds can eradicate cancer cells without any adverse effect [202]. There are discrepancies in the association of sex steroid hormone homeostasis and the ECS because it can enhance the growth rate of cancer cells, cell development, cell proliferation, and migration. Thus, for dynamic treatment purposes, the ECS has been considered a smart, innovative target in the initial period for pharmacological mediation in the fight against several hormone-related cancers.

### 6.9. Melanoma

Melanoma is mainly recognized as malignant cutaneous melanoma. It is a kind of skin cancer and the growing frequency of the highest mortality rate worldwide [206,207]. Such types of cancers can trace by great metastatic potential, enriched heterogeneity, and resistance to chemotherapy [208]. The availability of little groups of tumor cells known as melanoma initiating cells (MICs) is the critical aspect that is linked with failure treatment [209,210,211,212]. The ECS comprises endocannabinoid and their G-protein coupled receptors and enzymes responsible for their metabolism. The ECS performs a vital role in regulating signaling pathways involved in neoplastic transformation, tumor growth, and progression [8,213]. CB_1_ receptor modulation involves the administration of neurogenesis among the descent of neural crest melanoma cells and normal melanocytes [214].

In metastatic melanoma, usually, CNS is involved, and ECS may also perform function(s) in migration and tumor cell circulation in CNS [215]. It is suggested that AEA may induce cytotoxicity via triggering a caspase-dependent pathway in human melanoma cells [131,216]. In summary, the total cytotoxic effect of AEA occurs when a micro-molar range of concentrations is administrated and seems to be mediated through AEA by-products of COX-2 and LOX metabolism.

### 6.10. Basal Cell Carcinoma

Keratinocytes are the predominant entity available in stratified squamous epithelium. The propagation and variation of keratinocytes are essentially regulated and coordinated. On the lower membrane, the basal keratinocytes are bonded and indistinguishable but possess the ability to proliferate. Keratinocytes escape from the cell cycle before the differentiation pathway and migrate towards the epidermis surface. The migration towards the epidermis directed the development of the external layer of the epidermis. The constantly shaded external layer consisted of enucleated dead squamous from the skin surface [217]. The occurrence rate of both benign and malignant skin neoplasms has been significantly increased over the last few years. Therefore, non-melanoma skin cancer is one of the more frequent malignancies in humans. Basal cell and squamous cell carcinomas exhibit the enormous identified prevalence of malignant tumors [217,218,219,220]. These types of cancers are primarily developed from the hair follicle stem cells [218,221]. However, their growth and proliferation depend on initial burst neovascularization [222,223], in which obligatory entities are VEGF (vascular endothelial growth factor) [217,224] and EGFR (epidermal growth factor receptor) [218,222]. Moreover, the skin is an essential point for internal metastasis disease [217,218]. Patients associated with this disease can be controlled using early-stage recognition, validation by the biopsy, and appropriate therapy selection. Recently, different approaches are being used as cancer treatment therapies. Cryotherapy, such as 5-fluorouracil, a topical chemotherapeutic agent and photodynamic, although the molecules reduced penetration into the skin and make it difficult to enter into all cancer cells/tissues [218].

This particular mechanism encouraged to find whether (i), the skin as well as skin cancers possess receptors of cannabinoids; (ii), in vivo, the activation of cannabinoid receptor utilizes a role of growth-inhibition on skin cancers; (iii), and lastly, angiogenesis inhibition is associated with the anti-tumoral effect of cannabinoids. These findings illustrate (i), the presence of both receptors (CB_1_ and CB_2_) in the skin and skin cancer; (ii), in vivo activation of local cannabinoid receptor triggers the degeneration of skin tumors; (iii), and finally, tumor cells direct apoptosis and tumor angiogenesis inhibition mechanisms [225,226].

Recent research in ER stress-apoptosis shows the potential function of oxidative stress and whether this factor is controlled through receptors such as CB_1_, CB_2_, or TRPV1. In non-melanoma skin cancer cells (NMSC), the increasing level of intracellular glutathione and induced oxidative stress are both restrained by AEA. Trolox was employed to calculate the oxidative stress-induced via AEA, antioxidants, *N*-acetylcysteine (NAC) among cell death [225,226,227]. All antioxidants improved the anti-proliferative effect of AEA. Besides, the expression of CHOP10 is induced by AEA, and the Trolox inhibits the action of caspase three. Therefore, oxidative stress is required for AEA-induced ER stress-apoptosis. On the other hand, CB_1_, CB_2_, and TRPV1 did not inhibit the AEA-induced oxidative stress or ER stress-apoptosis. The AEA-induced ER-stress apoptosis in cells of NMSC is performed through oxidative stress by adopting the receptor-independent mechanism. In the future, to exclude NMSC receptor-independent mechanism, AEA signaling pathways can be rigorously analyzed or investigated [228,229,230].

### 6.11. Breast Cancer

The most communal type of cancer is breast cancer in women [231,232]. Identifying possible aspects that participate in the development of breast cancer is genetically linked to childbearing, lacking in breastfeeding, increasing levels of the hormone(s), and the deficiency of iodine [231,233,234]. The second name of breast cancer is malignant breast neoplasm. Malignant breast neoplasm derives from breast tissue, frequently from the milk ducts (carcinomas of the duct)/the lobules (carcinoma of lobule). Malignant breast neoplasm cells can reach different body organs (e.g., bones, lungs, and lymph nodes) [235,236]. Currently, the ECs system’s breast cancerfunction in regulatory pathways associated with tumor growth, induction of apoptosis, and administration of tumor vascularisation (angiogenesis) [12,202,231]. Angiogenesis is a process necessary for the transition of dormant state tumors to malignant state tumors [237,238].

The expression of CBR has been identified in tissues or cell lines of various breast cancer. The CB_1_ expression was tested using immunohistochemistry, and the result showed 14% of tumors in human breast cancer found in tissues stating the epidermal growth factor (EGF) family member. This is stated as the ErbB2 tyrosine kinase receptor. There was no association recorded among the expression of ErbB2 and CB_1_ [239]. In 28% of human breast cancer cells, the CB_1_ immunoreactivity was also expressed [240]. Different cell lines (i.e., T-47D, MCF-7, TSA-E1, MDA-MB-231, and MDA-MB-468) of breast carcinoma possess the CB_1_ receptors. The presence of CB_1_ receptors in human breast tissues was identified using different techniques; e.g., immunofluorescence, RT-PCR (real time polymerase chain reaction), and western blot analysis [196,241]. However, 72% of tissue of human breast carcinoma showed CB_2_ immunoreactivity. Surprisingly, CB_2_ receptors were seen within 91% of ErbB2-positive carcinoma tissues, suggesting an association between the expression of CB_2_ and ErbB2. However, no association was observed between the expression of CB_1_ and ErbB2 [239]. Another study revealed the detection of immunoreactivity of CB_2_ receptors in 355 human breast tumor cells [240]. Moreover, CB_2_ receptors were also observed using the RT-PCR, immunofluorescence, and western blotting methods in different human breast cancer cell lines and/or tissues [239,242,243,244,245]. RT-PCR technique used to detect the recognized subtype GPR55 of cannabinoid receptor was significantly expressed in breast cancer cell line (i.e., MDA-MB-231). However, a 30-fold lower level expression was recorded in MCF-7 breast cancer cell lines [246]. The recognized endogenous ligands (known as lysophosphatidylinositol (LPI)) for GPR55 perform triggering functions for the migration of cells and invasion in the carcinoma cell line MDA-MB-231. Specific LPI effect on migration is inhibited with the pretreatment of cannabidiol (CBD) [246]. It is elaborated that LPI activates the proliferation, and this effect is inhibited through CBD. The presence of hydrolyzing enzyme (FAAH) of anandamide in EFM-19 and MCF-7 carcinoma cell lines was detected through northern blotting analysis [247] or RT-PCR techniques [248]. Nevertheless, the available data recommends that subtypes of multiple CBR and catalyzing enzymes required for the hydrolysis of endocannabinoid (i.e., FAAH) exhibit a functional circulation suitable to proliferation, migration, and apoptosis of breast carcinoma cell regulation.

In vitro findings showed that ECs and cannabinoids-like compounds restrained proliferation and migration or apoptosis induction in various breast cancer cell lines. In some carcinoma cell lines, phytocannabinoid CBD facilitate the inhibition of cell proliferation [249,250,251,252], increase apoptosis activity [249], and decrease migration [250,251]. The fundamental mechanism of CBD in inducing such effects requires further study. Although, the anti-proliferative characteristic is the part of Δ^9^-THC [253,254,255], whereas increased apoptosis [256] and migration of carcinoma cell are restrained [241,257,258]. Anti-proliferative and pro-apoptotic actions are induced through Δ^9^-THC associated with the CB_2_ receptor [241,258,259].

**Figure 6:**
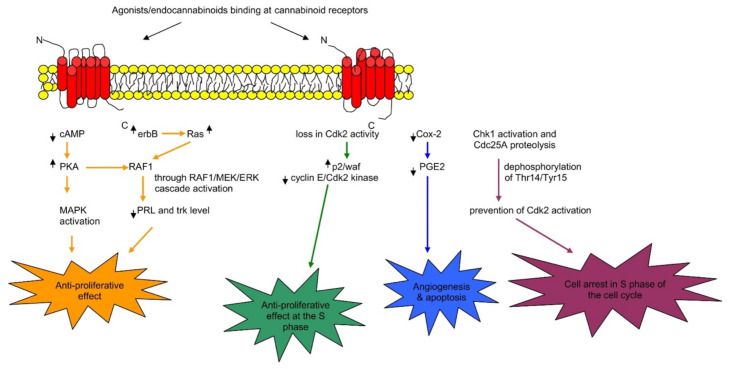
Schematic representation of examples of different pathways associated with anti-proliferative effects induced by cannabinoid receptor activation in breast cancer. Reprinted from Ref. [253] with permission from Elsevier. License Number: 5066110731347. Abbreviation: PKA (protein kinase A); cAMP (Cyclic adenosine monophosphate); MAPK (mitogen-activated protein kinase); Ras (rats sarcoma protein); Raf1 (proto-oncogene serine/threonine protein kinase); MEK (Mitogen-activated protein kinase); ERK (extracellular signal regulated kinase); PGE2 (prostaglandine E-2); TrK (tropomyosin receptor kinase); PRL (prolactin) COX-2 (cyclooxygenase-2); Cdk (cyclin-dependant kinase); Chk 1 (cell cycle checkpoint).

In vitro studies show the anti-proliferative properties linked with ECs. Cell proliferation certainly blocks AEA [253,260]; however, not through CB_2_ receptors. Although AEA and CB_2_ refereed effects may not be reduced, the individual study did not show their apparent. Furthermore, cell proliferation is blocked by 2-AG ole-amide, and arvanil, and their anti-proliferative effects are blocked by the CB_1_ (Figure 6) [253]. Remarkably, PEA that does not attach to CBR enhances the anti-proliferative effects of AEA, arvanil, olvanil and HU-210 [261], which is suggestive of a backup effect [262]. Additionally, AEA enhancement and the anti-proliferative effect of olvanil do not incorporate CB_2_ receptors. Methanandamide blocks both cell proliferation [261,263,264,265] and cell migration functions [264]. On the other hand, the mixture of a CB_1_ agonist (WIN55,212-2) and CB_2_ agonist (JWH-133) blocks the proliferation and migration of each cell [240,250]. This blockage pathway requires activation of CB_2_ receptors, but no data is available for the possible action of CB_1_. Remarkably, both agonists CP55 940 and HU-210 of cannabinoid [253] and antagonists SR141716A of cannabinoid CB [266] showed the inhibition of cell proliferation. Amide derivatives such as *N*-palmitoyl tyrosine and *N*-palmitoyl dopamine possess anti-proliferative characteristics [267]. Cell proliferation is blocked by the cannabidiol acid, cannabigerol cannabichromene, THC acid [253,262,267], and desacetyl levonantradol [252] in different breast carcinoma cell lines.

In vivo studies showed cannabinoids controlled the carcinoma growth, metastasis, cell proliferation, and angiogenesis determined using a mouse injected with various breast carcinomas cell lines. For instance, an animal model of metastatic breast cancer and metastases of the lung showed that Δ^9^-THC reduces the size, the number of cancer cells, and ErbB2 (tyrosine kinase receptor). Such cell proliferation inhibition involves the CB_2_ receptor but not the CB_1_ receptor [239]. The functions of CB_2_ receptors in anti-cancer properties are dependent on the CB_2_ agonist JWH-133 that can reduce carcinoma size and number [239,240]. Breast carcinoma cell proliferation is blocked through agonists of cannabinoids receptor of breast carcinoma cells via significant affinity nerve growth factor (Trk) and down-regulation of prolactin (PRL) receptors. Moreover, breast carcinoma susceptibility gene product (brca1) is down-regulated via the signaling pathways associated with CAMP-PKA/MAPK/Raf-ERK [268].

### 6.12. Cannabinoids in Liver Cirrhosis

In systemic or portal vasodilation and hypotension, ECs and CB_1_ receptors have been drawn in chronic liver cirrhosis [269,270]. The experimental data demonstrated that the CB_1_ receptor barrier, along with SR141716, overturned the hypotension and lowered the peripheral resistance. Moreover, the increased mesenteric flow of blood and portal pressure involve biliary, and carbon tetrachloride-induced cirrhosis is reduced among the rats. However, in non-cirrhotic administrated entities, these hemodynamic factors were modest through SR141716. Moreover, this data is specific and recommends an elevated endocannabinoid tendency in cirrhosis [270]. An elevated endocannabinoid tendency might support the up-regulation mechanism in CB_1_ receptors associated with hepatic vascular endothelial cells. This pathway may also facilitate the enhanced synthesis of anandamide through circulating monocytes. In the liver (from bile duct-ligated mice), elevated expression of CB_1_ receptors was recorded [271]. An elevated anandamide-induced vasorelaxation was exhibited in mesenteric arteries sequestered from cirrhotic of administered rats through CB_1_ and TRPV1 receptors [271,272]. The function of liver losses in cirrhosis due to the progressive elevation of endotoxin levels in plasma [271] is such an effect that is possibly associated with the increased synthesis of ECs among plasma monocytes, cirrhotic animals, and patients’ platelets [270]. Current experimental results suggested improved signaling via myocardial CB_1_ receptors in the cirrhotic cardiomyopathy pathogenesis [270].

The ECS can also be associated with liver fibrosis pathogenesis after the end-stage cirrhosis vasculopathy. In vitro studies have currently identified that anandamide carries antifibrogenic effects by blocking stimulated hepatic stellate cells at low micromolar concentrations and their necrosis induction through CB_1_/_2_ TRPV_1_–independent mechanism(s) at higher concentrations [273]. In the CB_2_ knockout mice model, the induction of carbon tetrachloride was much higher in liver fibrosis than their wild-type strain [270]. In liver biopsy samples from active cirrhosis patients, the expression of CB_2_ receptors was significantly induced [274]. Moreover, in myofibroblasts, activation of the CB_2_ receptor stimulates the inhibition of size and apoptosis. Thus, during chronic liver conditions, hepatic stellate cell activation emphasizes the antifibrogenic role of CB_2_ receptors. As anticipated from the above outcomes, the chronic practice of marijuana has been linked with hepatotoxicity relatively more than hepatoprotection [275]. Furthermore, current epidemiological study findings revealed that regular intake/smoking marijuana is a potential threat for fibrosis progression within chronic hepatitis C infection patients [276]. The stimulated activation of the pro-fibrogenic role of the CB_1_ receptor is supported by the preliminary experimental work related to the development of liver fibrosis where carbon tetrachloride is triggered in mice [277,278]. These concluding results recommend the comprehensive role of CB_1_ receptors in cirrhosis pathogenesis and also suggest further possible remunerations from the therapeutic use of CB_1_ antagonists during chronic liver disease.

## 7. Concluding Remarks

Cannabinoids in numerous animal models’ studies are active inflammatory modulators. Both in vivo and in vitro studies revealed an immunosuppressive effect of cannabinoids. However, further studies are required to carry out on human models. Biological and histological anti-inflammatory changes were tested in clinical trials of the following autoimmune diseases: multiple sclerosis, RA, scleroderma, and type-1 diabetes. In humans, autoimmune inflammatory bowel diseases, development of pain, positive influence on sleep, and improved life quality were explored. In ulcerative colitis, cannabinoid therapy reduced diarrhea conditions. Cannabinoids specific subtype: cannabidiol possesses less affinity towards both CB_1_ and CB_2_. Moreover, particularly synthetic cannabinoids show greater binding affinity to the CB_2_ receptor(s). They organize immunomodulatory effects lacking being psychoactive. Consequently, in research, they offer a possible therapeutic agent for autoimmune disorders. Nonetheless, cannabinoids are identified as potential candidates having numerous considerable antagonistic effects such as impairment of memory. Grey matter volume and reduction of IQ levels are both associated with THC. Few disorders related to psychology are also stimulated via THC. Due to the dopaminergic pathways, THC stimulation shows involvement in drug addiction, perhaps directed to dependence and drug abuse. Therefore, more research is required to illustrate how cannabinols would be utilized, their optimal concentrations, and which cannabinoids subtype should be more effective with less or no side effects.
